# Genome-Wide DNA Polymorphism Analysis and Molecular Marker Development for the *Setaria italica* Variety “SSR41” and Positional Cloning of the *Setaria* White Leaf Sheath Gene *SiWLS1*

**DOI:** 10.3389/fpls.2021.743782

**Published:** 2021-11-11

**Authors:** Hui Zhang, Sha Tang, James C. Schnable, Qiang He, Yuanzhu Gao, Mingzhao Luo, Guanqing Jia, Baili Feng, Hui Zhi, Xianmin Diao

**Affiliations:** ^1^Institute of Crop Science, Chinese Academy of Agricultural Sciences, Beijing, China; ^2^State Key Laboratory of Crop Stress Biology for Arid Areas, College of Agronomy, Northwest A&F University, Yangling, China; ^3^Department of Agronomy and Horticulture, University of Nebraska–Lincoln, Lincoln, NE, United States

**Keywords:** *Setaria italica*, molecular marker, positional cloning, white leaf sheath, mutant

## Abstract

Genome-wide DNA polymorphism analysis and molecular marker development are important for forward genetics research and DNA marker-assisted breeding. As an ideal model system for Panicoideae grasses and an important minor crop in East Asia, foxtail millet (*Setaria italica*) has a high-quality reference genome as well as large mutant libraries based on the “Yugu1” variety. However, there is still a lack of genetic and mutation mapping tools available for forward genetics research on *S. italica*. Here, we screened another *S. italica* genotype, “SSR41”, which is morphologically similar to, and readily cross-pollinates with, “Yugu1”. High-throughput resequencing of “SSR41” identified 1,102,064 reliable single nucleotide polymorphisms (SNPs) and 196,782 insertions/deletions (InDels) between the two genotypes, indicating that these two genotypes have high genetic diversity. Of the 8,361 high-quality InDels longer than 20 bp that were developed as molecular markers, 180 were validated with 91.5% accuracy. We used “SSR41” and these developed molecular markers to map the white leaf sheath gene *SiWLS1*. Further analyses showed that *SiWLS1* encodes a chloroplast-localized protein that is involved in the regulation of chloroplast development in bundle sheath cells in the leaf sheath in *S. italica* and is related to sensitivity to heavy metals. Our study provides the methodology and an important resource for forward genetics research on *Setaria*.

## Introduction

Foxtail millet (*Setaria italica*) and its wild ancestor green foxtail (*Setaria viridis*) are annual diploid grass species. Foxtail millet is a significant minor cereal crop and a dietary staple in a wide area of northern China and India. According to FAOSTAT reports, foxtail millet combined with other millet crops grown in an area of 9,349,969 hectares in China and India produced over 12.5 million tons of food resources in 2019.^1^

*Setaria italica* has many distinctive characteristics, including (i) C_4_ photosynthesis and tolerance to drought and low-quality soils; (ii) small, high-quality reference genomes (Bennetzen et al., 2012), with well-organized bioinformatic databases^2^,^3^; (iii) a short lifespan, the ability to self-pollinate, and prolific seed production; (iv) easy management in the laboratory (easily grown in growth chambers, no-seed shattering, and short seed dormancy). Because of these advantages, *Setaria* has been proposed as a molecular genetic model plant not only for C_4_ biology but also for crop functional genomics analyses (Diao et al., 2014).

Positional cloning of functional genes is one of the research hotspots in plant functional genomics. It has been proved to be a reliable and powerful tool for novel gene identification in well-established model systems, such as Arabidopsis and rice (*Oryza sativa*). In recent years, we have made significant advances in making positional cloning available for *S. italica* (Li et al., 2016; Liu et al., 2016; Zhang et al., 2018a), an emerging model system for Panicoideae grasses. Positional cloning starts with mutant or designed germplasm resources. “Yugu1” as its full-available genome sequences (Bennetzen et al., 2012), steady growth performance, and wide adaptability is an ideal plant material for gene functional analysis. Our team has constructed a large-scale ethyl methylsulfone (EMS)-induced mutant library based on “Yugu1”, and this library has been used in *Setaria* functional genomics research (Li et al., 2016; Zhang et al., 2018a). “SSR41” is another foxtail millet variety that originated from South Korea (Jia et al., 2013). We propose that “SSR41” is an ideal genotype to match to “Yugu1” mutants to construct mapping populations, because “SSR41” is morphologically similar to, but highly genetically divergent from, “Yugu1”. To data, several genes of *S. italica* have been cloned using “SSR41” and the corresponding “Yugu1” mutant, including *DPY1* (Zhao et al., 2020), *SiAGL1b* (Liu et al., 2016), *SiSTL1* (Tang et al., 2019), *SiSTL2* (Zhang et al., 2018a), *SiYGL1* (Li et al., 2016), and *SiYGL2* (Zhang et al., 2018b). In addition, “SSR41” is an elite cultivar with high yields, good grain quality, resistance to diverse diseases, and wide local adaptability, so it will be useful for breeding and crop improvement.

At present, there is little genetic background and genome information available for “SSR41”. With the progress in next generation sequencing (NGS) technologies, obtaining genome variation information for various plants has become more efficient, accurate, and cost-effective (Slatko et al., 2018). Mamidi et al. (2020) used the Illumina Hiseq 2000 platform to sequence and *de novo* assemble 589 green foxtail accessions. Using presence-absence variation (PAV) and single nucleotide polymorphism (SNP) diversity analyses, they defined the population structure and evolutionary history of *S. viridis* in North America. Through a genome-wide association study (GWAS) of an agronomically important phenotype, they identified valuable loci-controlling seed shattering and a leaf angle in *Setaria* (Mamidi et al., 2020). Therefore, high-throughput resequencing and comparative genomics analyses of foxtail millet varieties can provide useful information for the *Setaria* model system.

Leaf color (including leaf blade color, leaf sheath color, and leaf ligule color) is a common and easily identifiable agronomic trait in crops. Isolation of leaf color mutants and identifying the key genes regulating this trait are of great importance for determining how chlorophyll metabolism and chloroplast development are regulated (Wu et al., 2007), and for understanding the mechanisms of photosynthesis and designing crops with high photosynthetic efficiency (Cui et al., 2019). In addition, leaf sheath color can be used as a visible morphological marker to allow breeders to identify authentic hybrids (Oshima et al., 2019). Several studies have explored the genetic control of leaf sheath color in rice and maize (*Zea mays*), and a few genes regulating leaf sheath color have been identified (Li et al., 2018; Hu et al., 2020). In *S. italica*, only one leaf sheath color gene has been fine-mapped and cloned (Bai et al., 2020), but this is insufficient to support further research on this trait.

The *CLT1* gene encodes a chloroquine-resistance transporter-like transporter. This gene has been characterized in *Arabidopsis* and rice (Lim et al., 2011). Homozygous mutants lacking CLT1 transporter activity are sensitive to heavy metals, such as arsenic (As) and cadmium (Cd), and show disrupted glutathione (GSH) homeostasis (Yang et al., 2016). In rice, β-glucuronidase (GUS) staining of an *OsCLT1* promoter-GUS transgenic line indicated that this gene is highly expressed in the leaf sheath, while subcellular localization analyses revealed that OsCLT1 localizes in chloroplasts (Yang et al., 2016). However, the role of *CLT1* in regulating chloroplast or leaf sheath development is unknown.

In this study, deep whole-genome resequencing was employed to re-sequence the genome of the important *S. italica* genotype “SSR41”. The genomic variations and DNA polymorphisms between “SSR41” and “Yugu1” were identified. Characterization of these detected SNPs, InDels, and structure variations (SVs) shed light on the genetic basis of the desirable traits of “SSR41”. More than 8,000 genome-wide molecular markers were developed, and 180 of them were validated by PCR. Using these newly developed markers, we mapped the novel white leaf sheath gene *SiWLS1* encoding a chloroquine-resistance transporter in foxtail millet by positional cloning. Our research provides useful genome resources for gene cloning and molecular breeding of foxtail millet and will be useful for the further development of *Setaria* as a model system.

## Materials and Methods

### Plant Growth and Investigation of Agronomic Traits

“Yugu1” and “SSR41” are two foxtail millet varieties deposited in the National Crop Gene Bank, Chinese Academy of Agricultural Sciences (CAAS, Beijing, China). There are no segregating variants within each cultivar so that any sequence variations identified can confidently be interpreted as between-accession polymorphisms. A white leaf sheath mutant *siwls1* was isolated from the foxtail millet EMS mutant resource. The leaf sheath is white in the mutant but green in wild-type plants. An F_2_ mapping population of 982 individuals was generated by hybridization between “*siwls1*” and “SSR41”, and was used for map-based cloning. All plants were grown at the Shunyi Experimental Station at the CAAS (40°18′ N, 116°58′ E) during June 20 to October 5 in 2018. Five uniformly developed plants were collected at the seed-milking stage to investigate major agronomic traits. The assessment methods and scoring standards for agronomic traits have been described in our previous study (Jia et al., 2013).

### DNA Isolation and Whole Genome Resequencing

The genomic DNAs were extracted from leaves of 4-week-old plants of “SSR41”, “Yugu1”, the *siwls1* mutant, and individuals in the F_2_ population using a modified CTAB method (Zidani et al., 2005). Construction of DNA libraries and whole-genome resequencing were performed according to the Illumina DNA Prep workflow^4^ by the Beijing Berry Genomic Co. Ltd. (Beijing, China). The Illumina Hiseq 2500 platform and the 150-bp paired-end mode were employed for high-throughput sequencing. Raw sequencing reads have been submitted to the EMBL-EBI^5^ under the accession No. PRJEB46028. The reference genome was *S. italica* genome version 2.2.^6^ Reads filtering, reads mapping, variant calling, and variant annotation were performed as described in our previous study (He et al., 2021).

### Development and Validation of Polymorphic Molecular Markers

Previous studies have developed a number of simple sequence repeat (SSR) and SNP markers that can be used for genotyping different foxtail millet accessions (Jia et al., 2013; Zhang et al., 2014). However, SSR molecular markers need to be detected by denaturing polyacrylamide gel electrophoresis, which is laborious and time-consuming. Because of their codominance, convenience, practicality, and highly polymorphic nature (Jain et al., 2019), InDel markers, especially those longer than 20 bp, are becoming a popular agar-gel-based genotyping solution. Based on the resequencing results of “SSR41”, putative polymorphic InDels with length ≥20 bp were selected to develop molecular markers for foxtail millet. To further ensure the efficiency and polymorphism of the developed markers, we compared them among 1,826 *Setaria* accessions (1,237 *S. italica* and 589 *S. viridis*) for which NGS data are available (Jia et al., 2013; Mamidi et al., 2020; Li et al., 2021). The polymorphism information content (PIC) value of each marker was calculated as described elsewhere (Lemos et al., 2019). Primers were designed using Primer3 software^7^ and were used to amplify the sequences containing the putative polymorphic InDel loci. The optimized parameters for Primer3 inputs were as follows: PCR target product size, 200–350 bp; primer size, 18–29 bp; primer melting temperature (Tm), 55–64°C; and primer GC content, 35–65%. The primers designed for all molecular markers are listed in Supplementary Table 1. To validate the polymorphic molecular markers, we selected 180 markers evenly distributed among all nine chromosomes of *S. italica*. The target sequences were amplified by PCR using “SSR41” and “Yugu1” genomic DNA as the templates. The PCR products were separated in 3.0% agarose gels, stained with Gelred plus (mei5bio, Beijing, China), and imaged using the Gel Doc XR system (Bio-Rad, Hercules, CA, United States).

### Positional Cloning of *SiWLS1* Using the Newly Developed Molecular Markers

We used the F_2_ mapping population of 982 individuals obtained from hybridization between “*siwls1*” and “SSR41” for positional cloning. Fifty-four InDel markers evenly distributed across the nine chromosomes of foxtail millet (Supplementary Tables 1, 2) were selected for primary mapping using bulked segregant analysis (BSA). The basic principles and methods of BSA have been described previously (Michelmore et al., 1991), but a brief introduction to the method is given in Supplementary Figures 1A,B. In practical terms, we selected 30 recessive individuals segregated from the “SSR41” × “*siwls1*” F_2_ population. Then, DNA was extracted from each individual and mixed in equal amounts to generate a DNA pool. We used DNA from male (♂), female (♀), and F_1_ plants and the DNA pool (P) as templates for PCR amplifications of all selected markers (Supplementary Figures 1C,D). For fine mapping, five new InDel markers and three SNP markers located in the candidate region were developed, and 237 recessive individuals were analyzed. Candidate genes located within the finely mapped region were amplified and sent for Sanger sequencing to determine the mutation site. The molecular markers and primer sequences used for gene mapping and sequencing are listed in Supplementary Table 2. The phylogenetic tree of the candidate gene *SiWLS1* was constructed using MEGA X software with the maximum likelihood method and default parameters. The full-length protein sequences of SiWLS1 and its homologs used in this analysis are listed in Supplementary Table 3.

### Treatment and Phenotyping of the *siwls1* Mutant

To investigate the As and Cd sensitivity of *siwls1*, seeds of the mutant and wild type were germinated on moist filter paper overnight, and then exposed to solutions containing Na_3_AsO_4_ or CdSO_4_ at a range of concentrations (0, 10, 30, and 60 μM), respectively. Plants were grown in a growth chamber under a 10-h light (30°C)/14-h dark (26°C) photoperiod, with 40% humidity. After 7 days of treatment, five uniformly developed seedlings in each treatment were selected for measurements of plant height and root length. To investigate whether the white leaf sheath trait is related to chlorophyll (Chl) content, we measured Chl a and Chl b contents using acetone as the solvent. For these analyses, leaf sheaths of *siwls1* and Yugu1 plants were collected at the heading stage (*n* = 15). Details of the chlorophyll measurement method have been provided in our previous study (Li et al., 2016).

### Chloroplast Ultrastructure Observation and Subcellular Location Analysis

Fresh leaf sheaths of *siwls1* and “Yugu1” plants were collected at the stem elongation stage. Samples were cut into 2 × 1-mm^2^ sections and fixed in 2.5% glutaraldehyde overnight. The plant tissues were dehydrated, embedded, and cut into sections as described previously (Zhang et al., 2018a). Ultrathin sections of embedded samples were stained with uranyl acetate, and then observed using a transmission electron microscope (TEM) (JEM 1230, JEOL, Tokyo, Japan). To investigate the subcellular localization of SiWLS1 in foxtail millet, we constructed an SiWLS1-GFP fusion protein. The primers used for vector construction were as follows: 5′-gacgatatctctagaggatccATGGCGCCGCCGTCCCCT-3′ and 5′-gcccttgctcaccatggatccGTCATTCTTGTTGTGGAAAGAATTT C-3′, where lowercase sequences are adaptors containing *Bam*HI cleavage sites for the ClonExpress^®^ Ultra One Step Cloning Kit (C115-02, Vazyme Biotech, Nanjing, China). The fusion vector and the control vector were then transfected into foxtail millet protoplasts (Liu et al., 2016). Then, GFP fluorescence was observed and photographed under a Zeiss LSM 700 confocal microscope (Carl Zeiss, Jena, Germany).

## Results

### “SSR41” Is an Ideal Material to Coordinate With “Yugu1”

**FIGURE 1:**
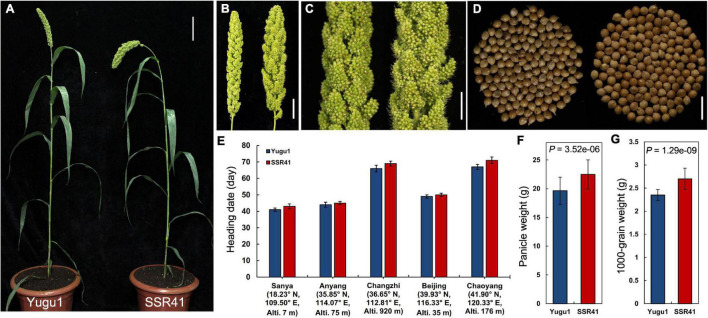
Phenotypes and statistical comparisons between “SSR41” and “Yugu1”. **(A)** Phenotype of whole plants at the heading stage. Bar = 10 cm. **(B)** Panicle of “Yugu1” (left) and “SSR41” (right). Bar = 3 cm. **(C)** An enlarged view of primary branches and spikelets of “Yugu1” (left) and “SSR41” (right). Bar = 1.5 cm. **(D)** Seeds of “Yugu1” (left; 150 seeds) and “SSR41” (right; 150 seeds). Bar = 5 mm. **(E–G)** Statistical comparisons of heading date, panicle weight, and 1,000-grain weight between “Yugu1” and “SSR41”. Values are means ± SD (*n* = 3 for heading date, *n* = 6 for panicle weight, and 1,000-grain weight). *P* values were determined using Student’s *t*-test.

Map-based cloning has become feasible for *S. italica* with the release of the “Yugu1” reference genome (Bennetzen et al., 2012) and the construction of a large-scale EMS-induced mutant library based on “Yugu1” (Sun et al., 2019). On that basis, we screened another *S. italica* germplasm “SSR41”, which readily cross-pollinates with “Yugu1”, and constructed large populations for mutation mapping. In the comparison between “Yugu1” and “SSR41” plants grown in different environments, major agronomic traits including mature plant height, leaf length and width, and panicle shape showed no significant differences (Supplementary Table 4 and Figures 1A–D). Therefore, the two varieties have similar plant stature and vegetative phenotypes, making it easy to identify mutant phenotypes in their hybrid progeny easily and clearly. Importantly, the heading dates and flowering periods of “SSR41” were almost identical to those of “Yugu1” in five different geographic regions (Figure 1E). These data indicate the two varieties are widely adapted to different environments, which is an advantage for designing cross-pollination experiments.

The values of agronomic characters related to crop yield, such as panicle weight and 1,000-grain weight, were significantly higher in “SSR41” than in “Yugu1” (Figures 1F,G), suggesting that “SSR41” is an ideal germplasm resource for breeding to improve foxtail millet.

### Whole Genome Resequencing of “SSR41”

For the above reasons, the *S. italica* genotype “SSR41” was chosen as a suitable partner with “Yugu1” for positional cloning of genes in *Setaria*. Hence, we used an NGS strategy for genome-wide detection of DNA polymorphism between the two genotypes. General statistics of the high throughput sequencing and reads mapping are shown in Supplementary Figure 2A. In total, 149,179,426 high-quality clean reads (>22.11 billion nucleotides) were generated. About 99.22% of the sequencing reads were successfully mapped onto the “Yugu1” reference genome. The average sequencing depth was approximately 48-fold, and the mapped sequences covered ∼95.73% of the reference genome. Analysis of the distribution of sequence coverage suggested that reads were evenly distributed throughout the available genome, indicating that the quality and randomness of our resequencing data were acceptable (Supplementary Figure 2B). The number of sequencing reads mapped to each SNP and the genomic distance between adjacent SNPs was calculated (Supplementary Figure 2C), and provided further evidence for the credibility of variation calling.

### Detection and Distribution of Variations Between the Two *S. italica* Genotypes

After filtering, 1,102,064 reliable SNPs were detected between “Yugu1” and “SSR41”, with a low heterozygosity ratio of 21.44%. On the basis of the nucleotide substitutions, these SNPs were divided into two categories: transitions (A↔G and T↔C) (1,400,101 transitions); and transversions (C↔A, G↔C, A↔T, and T↔G) (513,280 transversions), with an overall transition (Ts)/transversion (Tv) ratio of 2.73 (Supplementary Figure 3). We also evaluated the genome-wide InDel polymorphisms. In total, 196,782 InDels were identified after aligning sequences of “SSR41” and “Yugu1”, of which 162,262 (82.46%) were homozygous. The length of InDels ranged from 1 to 298 bp. Almost half of these variations (48.07%) were single-nucleotide InDels, 44.34% were 2–20 bp in length, and a small proportion (6.70%) was longer than 20 bp.

**FIGURE 2:**
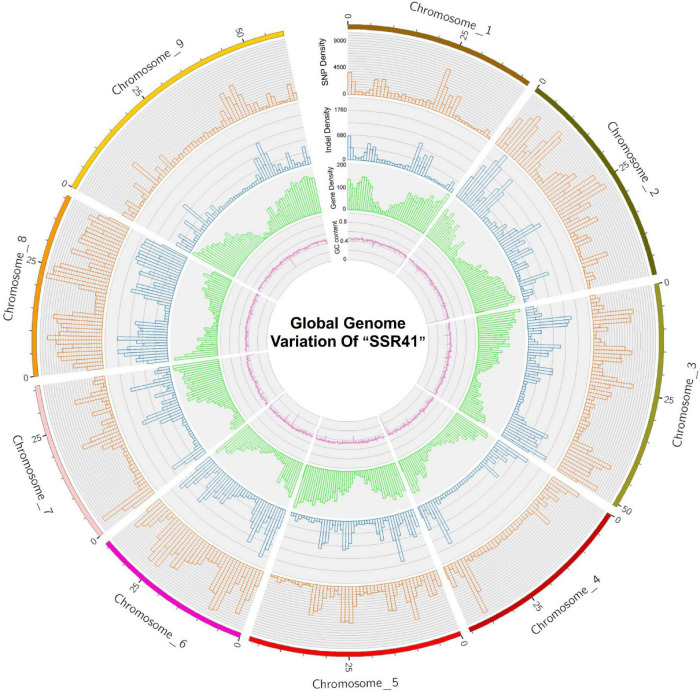
A circular plot showing “SSR41” genome sequence features. An orange bar shows single nucleotide polymorphism (SNP) density, a blue bar shows insertion/deletion (InDel) density, a green bar shows gene density, and a purple line shows GC content.

The average densities of polymorphisms detected between the “SSR41” and “Yugu1” genomes were 2719.65 SNPs per Mb, and 485.61 InDels per Mb. The average variant rates were one SNP every 368 bases, and one InDel every 2,059 bases. The distribution of SNPs and InDels varied among chromosomes. As shown in Supplementary Figure 3 and Figure 2, Chromosome 8 had the highest density of SNPs (5440.81 Mb^–1^) and InDels (982.08 Mb^–1^), and Chromosome 9 had the lowest SNP/InDel density (1333.66/240.74 Mb^–1^). We also calculated the numbers of SNPs/InDels per Mb on different chromosomes and found that the distribution of SNPs/InDels was uneven within each chromosome (Figure 2). High-density regions (12.8%) had >6,000 SNPs per Mb (e.g., 10,403 SNPs distributed on Chromosome 8, 22.0–23.0 Mb region), while four low-density regions had <200 SNPs per Mb (e.g., 136 SNPs on Chromosome 2, 44.0–45.0 Mb). The trends in the variant rates and distribution of InDels were similar to those of SNPs (Figure 2).

### Characteristics of SNPs, InDels, and SVs

**FIGURE 3:**
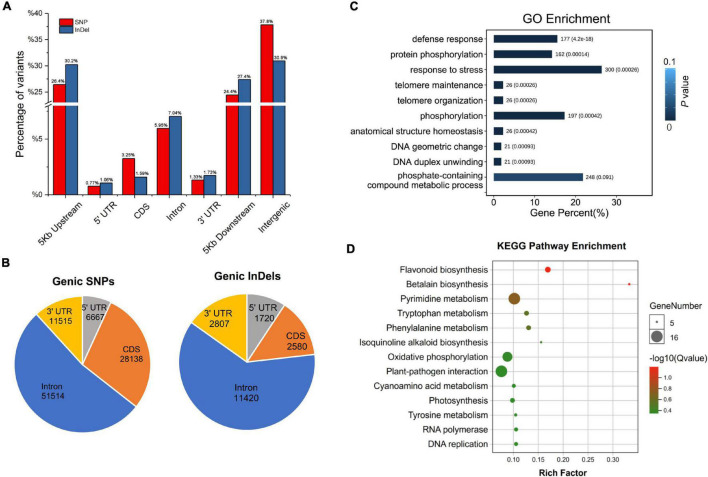
Annotation and functional enrichment analysis of “SSR41” genome variations. **(A)** Distribution of SNPs and InDels at the whole genome level. **(B)** Distribution of SNP and InDels in genic regions. **(C)** Gene ontology enrichment analysis of genes with large-effect variations. **(D)** Kyoto Encyclopedia of Genes and Genomes pathway enrichment analysis of genes with large-effect variations.

Among all the SNPs and InDels identified, only homozygous variations were collected for further annotation, because both “Yugu1” and “SSR41” are stable modern cultivars without phenotypic and genetic segregation. Of the 865,781 homozygous SNPs in “SSR41”, SNPs located in intergenic regions accounted for the largest proportion (37.82%), followed by those in regions 5 Kb upstream (26.44%) or downstream (24.44%) of coding regions (Figure 3A). Only 11.30% (97,833) of the SNPs were in genic regions. Of the 97,833 genic SNPs, just over half (51,514, 52.66%) were in introns (Figure 3B). Only 6.64% (3,418) of the intronic SNPs were located at splice regions, where they may change the original splicing pattern. There were 28,138 SNPs located in coding sequence (CDS) regions, 12,894 (45.82%) of which were synonymous SNPs, and 15,224 (54.18%), of which were non-synonymous variants. The ratio of non-synonymous to synonymous SNPs was about 1.18 across all gene models. We also identified that 6.81% (6,667) and 11.77% (11,515) of the SNPs occurred in the 5′ and 3′ untranslated regions. Annotation of InDel polymorphisms revealed that the general trends in the presence of 162,261 homozygous InDels in different genomic regions were similar to those of SNPs. Interestingly, there was a smaller proportion of InDels (1.59%) than SNPs (3.25%) in CDS regions, but a much higher proportion of InDels than SNPs in introns and gene upstream/downstream regions (Figure 3A).

We also identified SVs between “SSR41” and “Yugu1”, including 388 insertions (INS), 1,197 deletions (DEL), and 18 inversions (INV) with fragment sizes larger than 100 bp. Analysis of the distribution of the fragment sizes of these SVs revealed that 65.5% of them ranged from 100 to 400 bp in length (median, 311 bp) (Supplementary Table 5).

### Annotation and Effect of Genomic Variations on Functional Genes

Characterization of polymorphisms between “SSR41” and “Yugu1” revealed that approximately 89% of the variations were intergenic, while only 11% were genic. Among the genic variations, 3,979 polymorphisms were identified to have strong effects on gene function (including genomic variations leading to frame shifts, changes in splicing, loss of the start codon, and gain or loss of the stop codon) between the two accessions (Supplementary Table 6). The GO enrichment analysis showed that 10 subcategories in the “biological process” category, including defense responses (GO: 0006952), protein phosphorylation (GO: 0006468), and response to stress (GO: 0006950), were enriched with strongly affected genes (Figure 3C). The KEGG pathway enrichment analysis suggested that these genes were mainly related to 14 pathways, including secondary metabolite biosynthesis (e.g., flavonoid, tryptophan, and phenylalanine), oxidative phosphorylation, and plant-pathogen interaction (Figure 3D). Further characterization of these genes may shed light on the molecular basis of the phenotypic variations between “Yugu1” and “SSR41”.

### Genome-Wide Development and Validation of Polymorphic Molecular Markers

**FIGURE 4:**
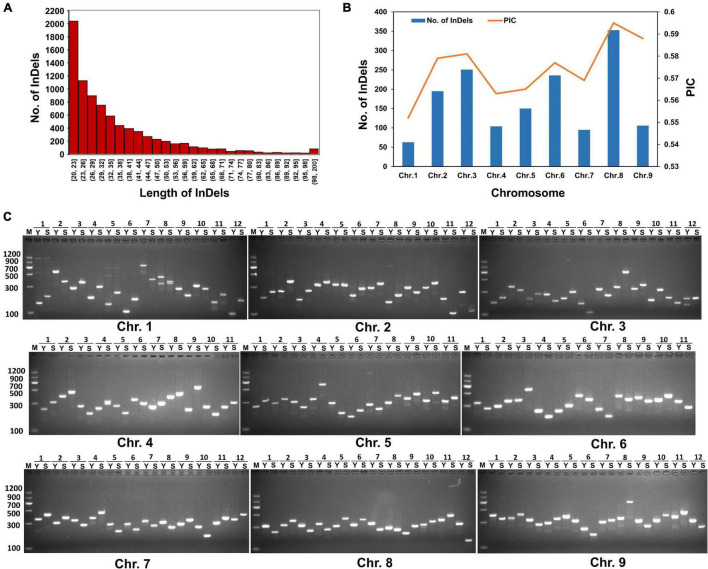
Characterization of polymorphic molecular markers. **(A)** Length distribution of InDel markers. **(B)** Polymorphism information content (PIC) variations of polymorphic molecular markers among different chromosomes. **(C)** Agarose gel electrophoresis analysis of PCR products amplified using representative molecular markers. M, DL2000 DNA marker. Y, PCR products amplified from “Yugu1” DNA as a template. S, PCR products amplified from “SSR41” DNA as a template. Numbers on top of each gel images indicate different molecular markers chosen from each chromosome.

Based on Indels and SVs identified from “SSR41” and “Yugu1”, we screened out high-quality variations that were 20 to 200 bp in length. These variations could be good candidates to design molecular markers that can be conveniently examined by PCR and DNA agarose gel electrophoresis. In total, 8,361 molecular markers evenly distributed on the nine chromosomes of foxtail millet were identified. Chromosome 8 had the largest number of InDels (1,781), followed by Chromosomes 2 (1,315) and 6 (1,287). Chromosome 1 had the fewest InDels (372). Half of these InDels had lengths of 20–32 bp (Figure 4A). To develop efficient and highly polymorphic InDel markers, the PIC values of these markers were characterized by comparison with 1,826 *Setaria* accessions, for which genome resequencing data are available (Jia et al., 2013; Mamidi et al., 2020; Li et al., 2021). In total, 1,551 highly polymorphic InDel markers with a PIC score higher than 0.5 were selected (Supplementary Table 1). The polymorphism levels and the number of these InDels were highest on Chromosome 8, followed by Chromosomes 3, 2, and 6. Then, 180 of these molecular markers were validated by PCR. More than 86% (156) of them generated PCR products, and 127 of them showed the expected polymorphism (Figure 4). These results confirmed that we had successfully developed polymorphic InDel markers for *Setaria*.

### Map-Based Cloning of a White Leaf Sheath Gene *SiWLS1* in *Setaria* Using the Newly Developed Molecular Markers

**FIGURE 5:**
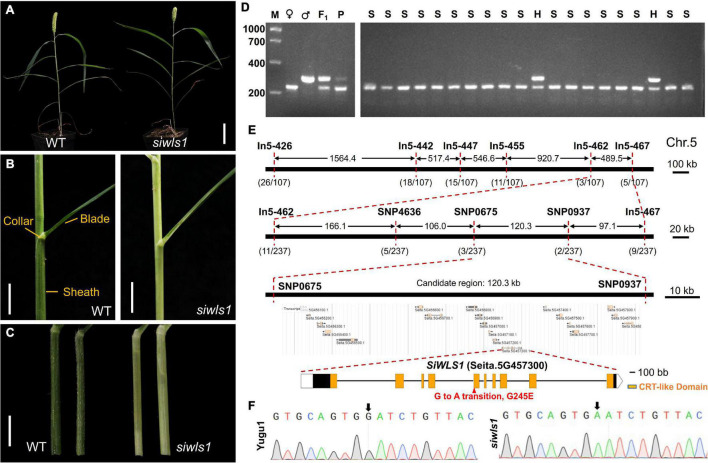
Characterization of foxtail millet white leaf sheath mutant *siwls1* and identification of the candidate gene. **(A)** General statures of *siwls1* mutant and wild-type plants at maturity. Bar = 5 cm. **(B)** Leaf blade, leaf collar, and leaf sheath morphology of “Yugu1” (WT) and *siwls1*. Bar = 1 cm. **(C)** An enlarged view of leaf sheath of wild-type and mutant plants. Bar = 1 cm. **(D)** An example of a molecular marker “In5-455” closely linked to the *SiWLS1* locus. M, DL 2000 molecular weight marker; ♀, PCR product amplified from female parent *siwls1*; ♂, PCR product amplified from male parent SSR41; F_1_, PCR product amplified from F_1_ individual; P, PCR product amplified from the DNA pool of a mixture of 30 recessive homozygous individuals in F_2_ population; S, PCR product amplified from recessive homozygous individual in F_2_ population; H, PCR product amplified from heterozygous individual in F_2_ population. **(E)** Positional cloning of *SiWLS1* using newly developed molecular markers. **(F)** Sanger sequencing validation of the causal SNP.

A *S. italica white leaf sheath* mutant *siwls1* was screened out from the “Yugu1” EMS mutant library. Analyses of its agronomic traits indicated that the mature plant height, panicle length, panicle diameter, length, and width of the leaf blade of *siwls1* were not significantly different from those of wild-type plants (Figure 5A and Supplementary Table 7). From the heading stage to the mature stage, the *siwls1* mutant exhibited a white leaf sheath, different from the green leaf sheath of “Yugu1”, while the leaf collar and leaf blade were similar to those of wild type (Figures 5B,C).

For the genetic analysis of the *siwls1* mutant and fine mapping of its candidate gene, we constructed an F_2_ population from hybridization between *siwls1* and “SSR41”. All individuals in the F_1_ generation displayed a green leaf sheath, the same as that of wild-type plants. In the F_2_ generation, the segregation ratio of plants with a green leaf sheath (745 individuals) and those with a white leaf sheath (237 individuals) was close to 3:1 (Supplementary Table 8). These results clearly indicated that the white leaf sheath phenotype is controlled by a single recessive nuclear gene.

Using 30 F_2_ homozygous recessive individuals and 54 newly developed InDel markers, we found that the target gene was located on the long arm of Chromosome 5 around the marker In5-455 using the BSA method (Supplementary Figures 1C,D and Figure 5D). For fine mapping, we further developed five InDel markers and three SNP markers, and used them to genotype all 237 recessive individuals (Figure 5E). Our gene mapping analyses eventually limited the target gene to a 120.26-kb region on Chromosome 5 between the markers SNP0675 and SNP0937 (Figure 5E). According to the foxtail millet reference genome annotation^8^ (*S. italica* v2.2), there are 19 genes within this region. To identify which one is responsible for the white leaf sheath phenotype, we performed whole genome re-sequencing of four DNA pools (from mutants, wild-type plants in the F_2_ population, and both parents). In a BSA sequencing (BSA-seq) analysis, only one SNP (G to A at 46,571,596 bp on Chromosome 5) met the certification standard (as described in Supplementary Table 9). Sanger sequencing and PCR analysis confirmed the presence of a G-to-A transition in the fifth exon of *Seita.5G457300*, leading to an amino acid change from Gly-245 to Glu in the chloroquine-resistance transporter (CRT)-like domain (Figures 5E,F). These results revealed that *Seita.5G457300* corresponds to *SiWLS1*.

### *SiWSL1* Encoding a Chloroquine-Resistance Transporter Affects Chloroplast Development and Chlorophyll Accumulation in the Leaf Sheath of Foxtail Millet

**FIGURE 6:**
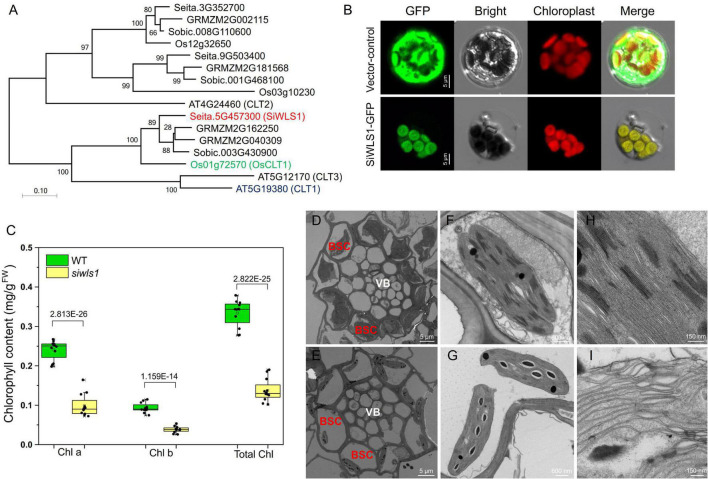
Functional analysis of foxtail millet white leaf sheath gene *SiWLS1*. **(A)** Phylogenetic relationship between SiWLS1 and its homologs in maize, sorghum, rice, and Arabidopsis. **(B)** Subcellular localization of SiWLS1 protein. **(C)** Comparison of chlorophyll contents between *siwls1* mutant and wild-type plants. **(D–I)** Chloroplast ultrastructure in siwls1 mutant (the lower row) and wild-type (the upper row) plants. VB, vascular bundle; BSC, bundle sheath cells.

Sequence analysis showed that *SiWLS1* is a single-copy gene with a 1,335-bp CDS encoding a 444-amino acid protein that belongs to the CRT-like family. A phylogenetic tree was constructed to examine the relationships of the *Setaria* CRT-like gene family with those of Arabidopsis, rice, maize, and *Sorghum bicolor*. Sixteen CRT-like genes were identified: three in *S. italica*, four in maize, three in *S. bicolor*, three in rice, and three in Arabidopsis (Figure 6A and Supplementary Table 3). The genes with the strongest similarity to *SiWLS1* in rice and Arabidopsis were *Os01g72570* (*OsCLT1*) and *AT5G19380* (*CLT1*), respectively, which are known to be key regulators of glutathione homeostasis and heavy metal tolerance (Lim et al., 2011; Yang et al., 2016). According to the results of a study on *OsCLT1* in rice (Yang et al., 2016), the *osclt1* mutant is hypersensitive to As and Cd. Therefore, we determined the sensitivities of *siwls1* toward As and Cd treatment. As shown in Supplementary Figures 4, 5, the root growth of *siwls1* was severely inhibited in response to increasing As and Cd concentrations compared with that of “Yugu1”, indicating that the *siwls1* mutant is more sensitive to As and Cd. These results were consistent with those of a previous study on rice (Yang et al., 2016), supporting that *SiWLS1* is the causal gene.

To determine the subcellular localization of the SiWLS1 protein, we fused the *SiWLS1* CDS to *GFP* and transiently introduced it into foxtail millet protoplasts. The SiWLS1-GFP signal was detected in chloroplasts, as determined from the clear overlap of the GFP signal with the autofluorescence of chloroplasts (Figure 6B), suggesting that SiWLS1 functions in chloroplasts. We measured the chlorophyll contents in the leaf sheath of the *siwls1* mutant and “Yugu1”, and found that the Chl*a*, Chl*b*, and total Chl contents were significantly lower in *siwls1* plants than in the wild type (Figure 6C). Ultrastructural observations of chloroplasts revealed dramatic reductions in the number and the size of chloroplasts in bundle sheath cells (BSC) in the *siwls1* mutant compared with “Yugu1” (Figures 6D,E). The stroma lamella and grana lamella of chloroplasts were highly ordered in wild type, while the thylakoids in *siwls1* chloroplasts appeared to be disorganized (Figures 6F–I). These findings, together with the white leaf sheath phenotype of *siwls1*, indicated that *SiWLS1* plays an important role in regulating chlorophyll accumulation or chloroplast development in the leaf sheath of foxtail millet.

## Discussion

### “Yugu1” and “SSR41” Are Ideal Resources for Forward Genetics and Map-Based Cloning Research in *S. italica*

The foxtail millet variety “Yugu1” is a modern cultivar that is widely grown in northern China. Because of its outstanding agronomic performance and its wide adaptability to different growth environments, “Yugu1” was selected to generate a high-quality *Setaria* reference genome (Bennetzen et al., 2012) and a large-scale EMS mutant library (Sun et al., 2019). To further promote the development of the “Yugu1”-based model for gene cloning, we screened another *S. italica* variety, “SSR41”. As expected, “SSR41” flowered at the same time as “Yugu1” and easily hybridized with it. Multi-year and multilocation comparisons of the two varieties have shown that “Yugu1” and “SSR41” are very similar in terms of their growth, development, and general plant stature. This ensures that the hybrid offspring of “SSR41” and “Yugu1” will have a steady growth performance, which is beneficial for the selection of individuals in an F_2_ mapping population.

We used high-throughput sequencing to obtain detailed genome sequence information for “SSR41”. The mean sequencing depth for “SSR41” was up to 48×, deeper than that of previous sequencing of *Setaria* accessions (Bai et al., 2013; Mamidi et al., 2020; Li et al., 2021). High-quality sequence reads of “SSR41” were aligned to “Yugu1” (the reference genome) to discover genome-wide polymorphic molecular markers. More than 1.2 million variations were identified in “SSR41”; more than the number of DNA variations detected in another foxtail millet variety, “SLX” (Bai et al., 2013). Interestingly, although “Yugu1” and “SSR41” are morphologically similar, these two varieties have abundant DNA polymorphisms. This has allowed us to develop sufficient molecular markers to support genotyping of the genetic population generated from “Yugu1” and “SSR41”.

Due to our continuing efforts, positional cloning in *Setaria* is now practicable using “SSR41” and “Yugu1” EMS mutants as plant materials. To date, map-based cloning has been used to characterize at least eight *S. italica* genes, including *SiMADS34* (Hussin et al., 2021), *DPY1* (Zhao et al., 2020), *SiSTL1* (Tang et al., 2019), *SiSTL2* (Zhang et al., 2018a), *SiYGL1* (Li et al., 2016), *SiYGL2* (Zhang et al., 2018b), *Loose Panicle1* (Xiang et al., 2017), and *SiAGO1b* (Liu et al., 2016). These studies illustrate that “SSR41” and “Yugu1” are suitable germplasm resources for forward genetics and positional cloning research on *Setaria*.

### Development of Polymorphic InDel Markers for the *Setaria* Model System

With the development of high-throughput sequencing, molecular marker development has become feasible and convenient for various crops. Both small InDels and SVs have become powerful marker systems for genotyping, SV-GWAS, and map-based cloning analyses (Qin et al., 2021). Previous studies on molecular marker development in *Setaria* have focused on SSR markers (Jia et al., 2007). To date, more than 2,000 effective SSR markers have been developed for genetic diversity analysis and quantitative trait loci mapping (Zhang et al., 2014). However, genotyping using SSR markers is laborious and time-consuming. In comparison, InDel markers, especially those longer than 20 bp, are better for genotyping because they can easily be detected by DNA electrophoresis on agarose gels. In the present study, we identified 8,361 InDels, and all of them were compared with genome variations of 1,826 *Setaria* accessions to evaluate their PIC values. Among them, 1,551 were highly polymorphic with a high-average PIC value of 0.579. Of the 180 tested pairs of primers for InDel markers, 94% of them produced stable PCR amplification products, and 127 of them were polymorphic (Figure 4C). These results confirmed that we have developed a large set of highly polymorphic InDel markers for *Setaria*. These markers are a valuable resource for positional cloning and other genotyping analyses in *Setaria*.

### Characterization of Genome Variations in “SSR41” Provides Insights Into the Genetic Basis of Its Preferable Agronomic Traits

The discovery and the utilization of genome variations, including SNPs, InDels, and SVs, form the basis of gene mapping and marker-assisted breeding. “SSR41” is an elite foxtail millet variety with many desirable agronomic traits such as high yield, disease resistance, and good grain quality. In this study, we performed whole-genome sequencing of “SSR41” and identified a number of high-quality SNPs, InDels, and SVs. Among these genome variations, only a few were identified to affect gene function. Functional enrichment analysis suggested that genes related to defense response and plant-pathogen interaction were significantly enriched with sequence variations. *Seita.7G239900*, which is homologous to *FLAGELLIN-SENSITIVE 2* (*FLS2*, *AT5G46330*), is one of the most important disease-resistance genes, and it had two insertion mutations in “SSR41”. FLS2 functions in the MAP kinase signaling pathway, which is involved in innate immunity (Gómez-Gómez and Boller, 2000). In Arabidopsis, the *fls2-17* mutant exhibits a significant flg22-induced reduction in bacterial growth compared with that in wild-type plants (Zipfel et al., 2004). Thus, the variation in *FLS2* in “SSR41” may contribute to its enhanced disease resistance. Interestingly, we detected mutations in some genes related to starch and sucrose metabolism, which may directly affect grain quality. Among these mutations, a C-to-A transversion leading to an early stop codon was identified at the second exon of *Seita.6G036700* in “SSR41”. *Seita.6G036700* is homologous to a soluble starch synthase IIIa (*OsSSIIIa*) in rice. The OsSSIIIa-deficient rice mutants have smaller and rounder starch granules compared with those of wild type and show reduced contents of long-chain starch in the endosperm (Ryoo et al., 2007). Thus, the mutation of this gene in foxtail millet “SSR41” may be related to its high-grain quality.

### *Setaria* Is an Efficient Model System for Exploring Novel Gene Function in Plant Biology

For decades, Arabidopsis has been widely used as a model plant for research on many aspects of biology and technology innovation; thus, it has been at the center of basic plant basic research. However, during their long evolutionary history, plants have evolved specific characteristics and systems, such as C_4_ photosynthesis. Thus, more model plants with a diverse range of characters are required to detect novel gene structures and functional variations. In recent years, *Setaria* has been proposed as an elegant C_4_ model because of its distinct growth and development characteristics. Numerous research groups have made efforts to improve *Setaria* as a model plant, including providing high-quality *Setaria* genome sequence data (Yang et al., 2020), improving its *Agrobacterium*-mediated transformation efficiency (Van Eck, 2018) and providing new technical platforms (Fahlgren et al., 2015). Our group has focused on exploring *Setaria* gene functions using forward genetics approaches. In this study, we developed a sufficient number of polymorphic InDel markers that can be used to improve map-based cloning efficiency in *Setaria*. Leaf sheath color is an important trait that can be used not only to study the molecular mechanisms that regulate chloroplast development but also as a practical morphological marker to assist breeding. A novel white-leaf sheath mutant *siwls1* was isolated. The wild-type plants have a green leaf sheath, but the mutant exhibits a white leaf sheath from the heading stage to the mature stage. The candidate gene *SiWLS1* was identified by analyzing 237 F_2_ recessive individuals with 65 InDel markers. The laboratory work took 2 months, demonstrating that map-based cloning of a specific gene locus in *Setaria* is feasible and efficient.

The results of many other studies and our present study suggest that the *Setaria* model can reveal new functions of previously characterized genes. *SiWLS1* is homologous to rice *OsCLT1*, which encodes a CRT-like transporter that is essential for glutathione homeostasis and As and Cd detoxification in rice (Yang et al., 2016). In the present study, we also found that the *Setaria siwls1* mutant was sensitive to As and Cd treatment, indicating that *CLT1* has a conserved function to regulate heavy metal accumulation and distribution in plants. However, as a chloroplast-located protein, CLT1 may also regulate chloroplast development and chlorophyll synthesis, although little is known about this role at present. Here, we identified a novel white leaf sheath *Setaria* mutant *siwls1* harboring a homozygous recessive mutation in the *CLT1* gene. Analyses by TEM revealed a defective chloroplast development phenotype in bundle sheath cells of the *siwls1* leaf sheath (Figures 6D–I). Additionally, the chlorophyll content was significantly lower in *siwls1* plants than in wild-type plants (Figures 6D–I), indicating that *CLT1* affects chloroplast development and chlorophyll accumulation in the leaf sheath of foxtail millet. Other studies have also reported new functions of genes determined using the *Setaria* model, for example, *Bristleless1 (Bsl1)* in *S. viridis*. Yang et al. (2017) identified two *bsl* mutants in *S. viridis*, both of which showed defective bristle development. Then, BSAseq identified the candidate gene, *BSL1*, which is homolog of *Dwarf11* (*D11*) in rice. D11 encodes a cytochrome P450 protein involved in brassinosteroid biosynthesis. Although *BSL1* and *D11* are syntenic orthologs, their biological function to regulate bristle and inflorescence development is unique in *Setaria*, and has not been reported for either Arabidopsis or rice (Yang et al., 2017). From the results of our study and other studies on gene function in *Setaria*, we can clearly demonstrate that both *S. italica* and *S. viridis* are ideal model systems for exploring novel gene functions in plants.

## Data Availability Statement

The original contributions presented in the study are publicly available. This data can be found here: EMBL’s European Bioinformatics Institute (EMBL-EBI) BioProject database under accession number PRJEB46028.

## Author Contributions

XD and ST conceived the project. HZa, ST, YG, ML, GJ, and BF conducted the experiments and collected the data. HZi constructed the mapping population. ST, JS, and QH conducted bioinformatic analysis. HZa and ST drafted the manuscript. XD and JS revised the manuscript. All the authors contributed to the article, read, and approved the manuscript.

## Conflict of Interest

The authors declare that the research was conducted in the absence of any commercial or financial relationships that could be construed as a potential conflict of interest.

## Publisher’s Note

All claims expressed in this article are solely those of the authors and do not necessarily represent those of their affiliated organizations, or those of the publisher, the editors and the reviewers. Any product that may be evaluated in this article, or claim that may be made by its manufacturer, is not guaranteed or endorsed by the publisher.

## Footnotes

1
http://www.fao.org

2
http://foxtail-millet.biocloud.net

3
http://www.setariamodel.cn

4
www.illumina.com

5
http://www.ebi.ac.uk/

6
phytozome.jgi.doe.gov/pz/portal.html#!info?alias=Org_Sitalica

7
https://github.com/primer3-org/primer3

8
http://phytozome.jgi.doe.gov/

## Supplementary Material

Supplementary Figure 1 Introduction to positional cloning in foxtail millet using “SSR41”. **(A)** A general view of positional cloning in foxtail millet. **(B)** Introduction of linked and unlinked markers used in PCR tests. **(C)** A summary of bulked segregant analysis (BSA) for primary mapping of *SiWLS1*. **(D)** DNA agarose gel electrophoresis results for primary mapping.

Supplementary Figure 2 General statistics of “SSR41” resequencing data. **(A)** Genome-wide distribution of read coverage. **(B)** Statistics of read coverage and sequencing depth. **(C)** Single nucleotide polymorphism (SNP) depth and neighboring SNP distance distributions.

Supplementary Figure 3 Annotations of SNPs between “SSR41” and “Yugu1”.

Supplementary Figure 4 Comparisons of plant growth between “Yugu1” and *siwls1* in response to arsenic treatment. Significant differences were determined by Student’s *t*-test (ns = no significant difference, ^∗^*p* < 0.01, ^∗∗^*p* < 0.001).

Supplementary Figure 5 Comparisons of plant growth between “Yugu1” and *siwls1* in response to cadmium treatment. Significant differences were determined by Student’s *t*-test (ns = no significant difference, ^∗^*p* < 0.01, ^∗∗^*p* < 0.001).
