# Supplementary material for: Genome-Wide DNA Polymorphism Analysis and Molecular Marker Development for the Setaria italica Variety “SSR41” and Positional Cloning of the Setaria White Leaf Sheath Gene SiWLS1
Source: Front Plant Sci. 2021 Nov 11;12:743782. doi: 10.3389/fpls.2021.743782 (PMC8632227; doi:10.3389/fpls.2021.743782)
Supplement: Supplementary Figure 1 — Introduction to positional cloning in foxtail millet using “SSR41”. (A) A general view of positional cloning in foxtail millet. (B) Introduction of linked and unlinked markers used in PCR tests. (C) A summary of bulked segregant analysis (BSA) for primary mapping of SiWLS1. (D) DNA agarose gel electrophoresis results for primary mapping. [file Presentation_1.PPTX]

## Slide 1
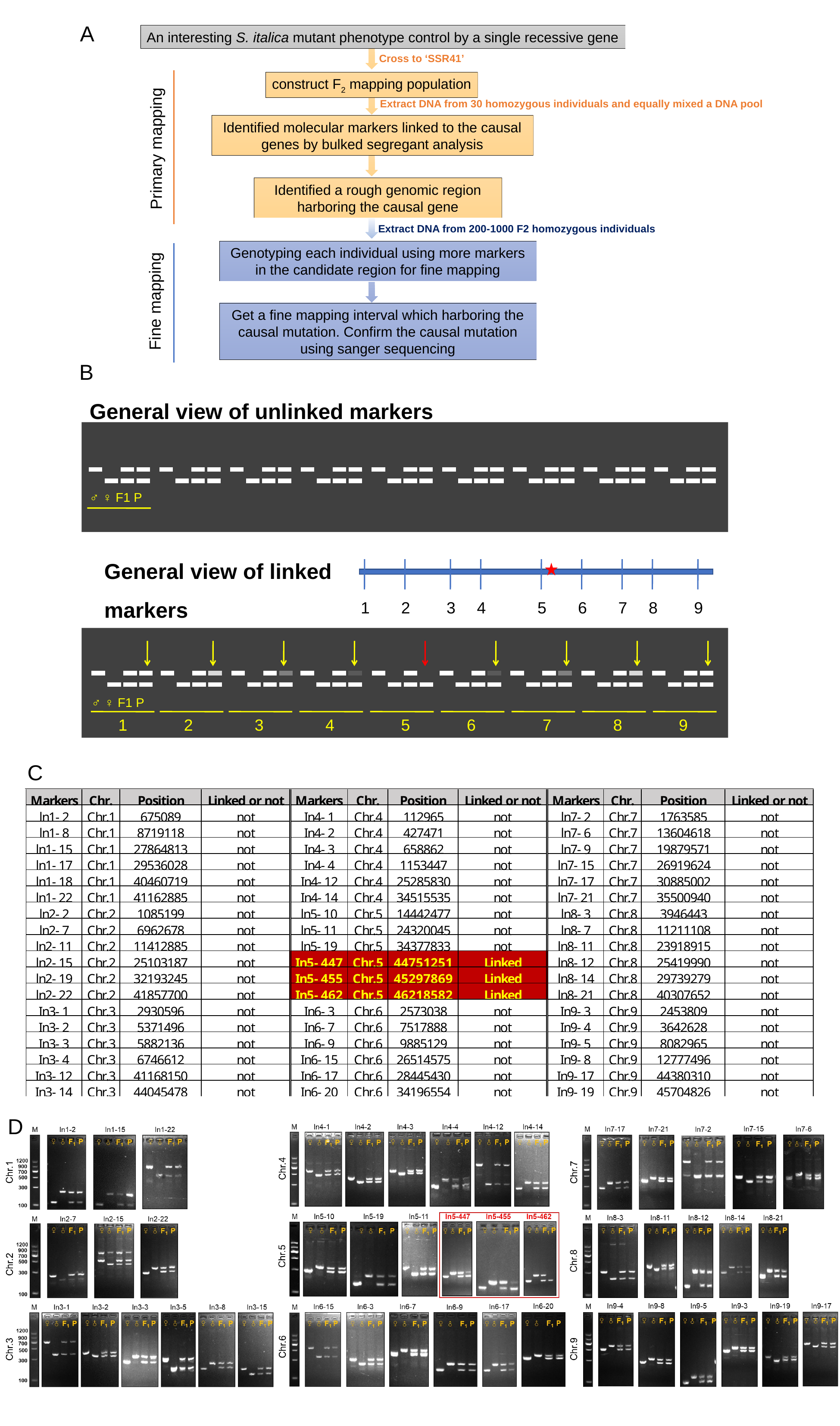

A
An interesting S. italica mutant phenotype control by a single recessive gene
Cross to ‘SSR41’
construct F2 mapping population
Extract DNA from 30 homozygous individuals and equally mixed a DNA pool
Identified molecular markers linked to the causal genes by bulked segregant analysis
Identified a rough genomic region harboring the causal gene
Extract DNA from 200-1000 F2 homozygous individuals
Genotyping each individual using more markers in the candidate region for fine mapping
Get a fine mapping interval which harboring the causal mutation. Confirm the causal mutation using sanger sequencing
Primary mapping
Fine mapping
B
General view of unlinked markers
♂ ♀ F1 P
General view of linked markers
1
2
3
4
5
6
7
8
9
♂ ♀ F1 P
1
2
3
4
5
6
7
8
9
C
D
