# Supplementary figures and images for: Genome-Wide DNA Polymorphism Analysis and Molecular Marker Development for the Setaria italica Variety “SSR41” and Positional Cloning of the Setaria White Leaf Sheath Gene SiWLS1
Source: Front Plant Sci. 2021 Nov 11;12:743782. doi: 10.3389/fpls.2021.743782 (PMC8632227; doi:10.3389/fpls.2021.743782)

## Slide 1
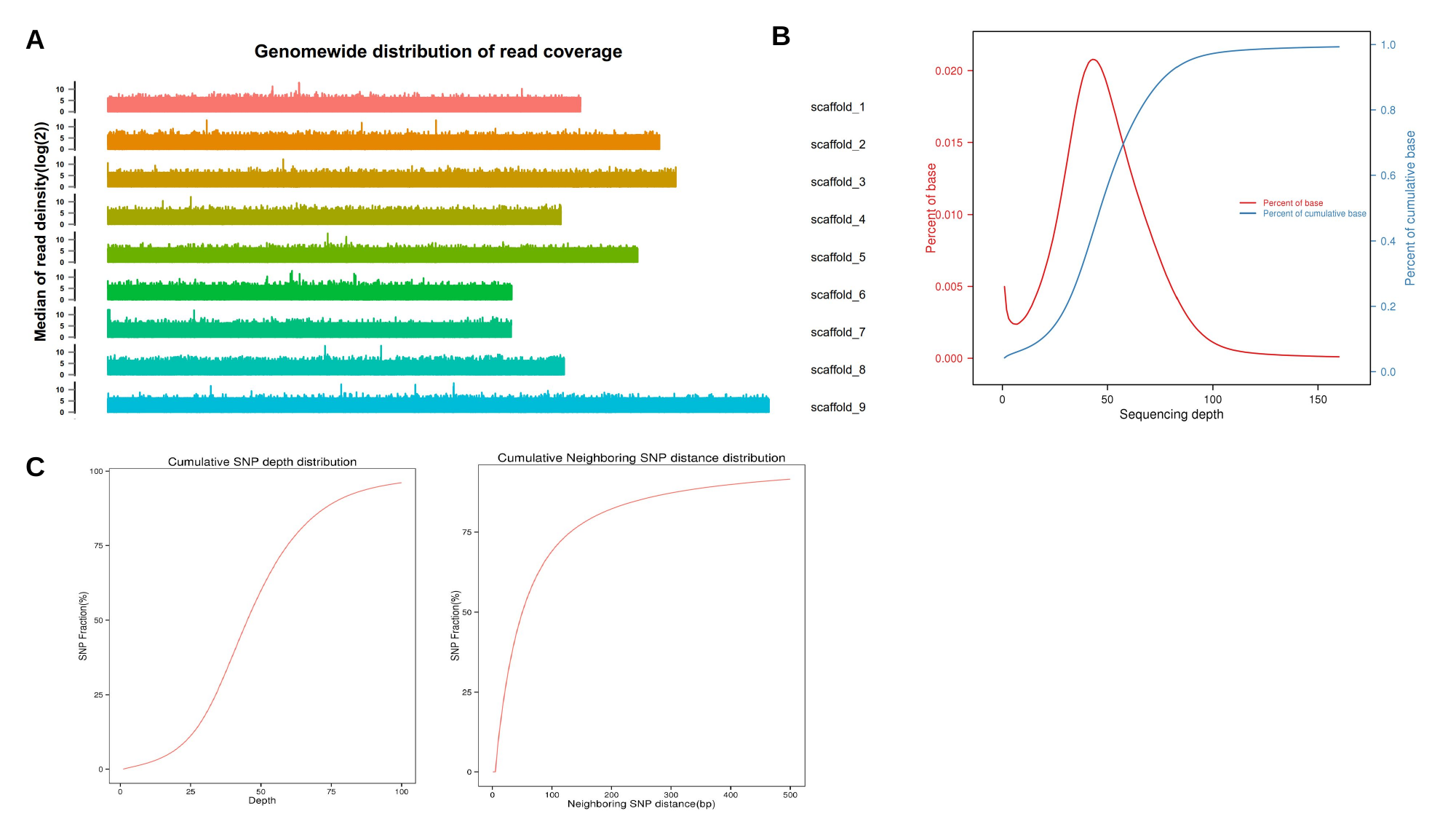

B
A
C

Supplement: Supplementary Figure 2 — General statistics of “SSR41” resequencing data. (A) Genome-wide distribution of read coverage. (B) Statistics of read coverage and sequencing depth. (C) Single nucleotide polymorphism (SNP) depth and neighboring SNP distance distributions. [file Presentation_2.PPTX]

## Slide 1
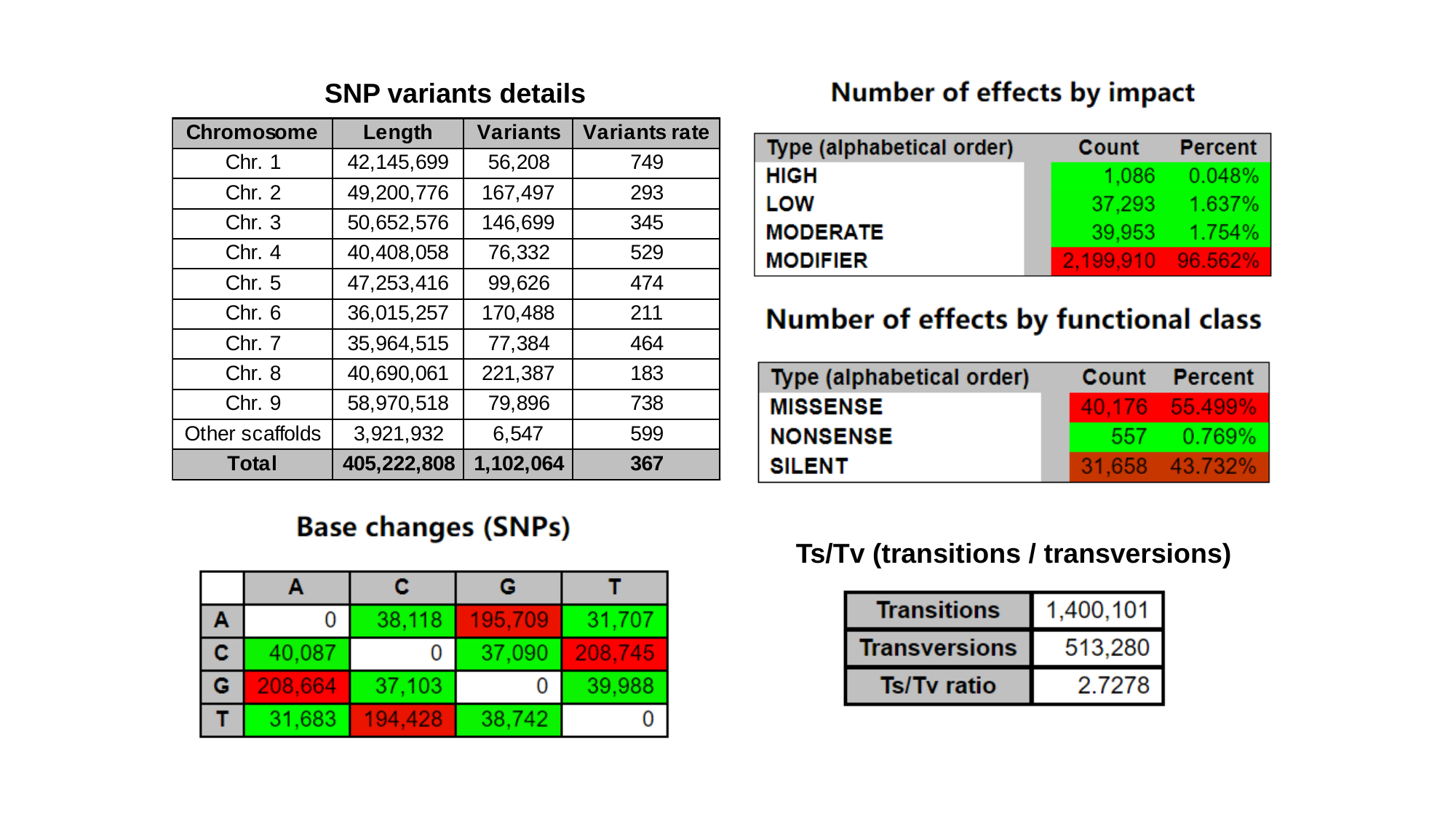

SNP variants details
Ts/Tv (transitions / transversions)

Supplement: Supplementary Figure 3 — Annotations of SNPs between “SSR41” and “Yugu1”. [file Presentation_3.PPTX]
