# Supplementary material for: Genome-Wide DNA Polymorphism Analysis and Molecular Marker Development for the Setaria italica Variety “SSR41” and Positional Cloning of the Setaria White Leaf Sheath Gene SiWLS1
Source: Front Plant Sci. 2021 Nov 11;12:743782. doi: 10.3389/fpls.2021.743782 (PMC8632227; doi:10.3389/fpls.2021.743782)
Supplement: Supplementary Figure 4 — Comparisons of plant growth between “Yugu1” and siwls1 in response to arsenic treatment. Significant differences were determined by Student’s t-test (ns = no significant difference, ∗p < 0.01, ∗∗p < 0.001). [file Presentation_4.PPTX]

## Slide 1
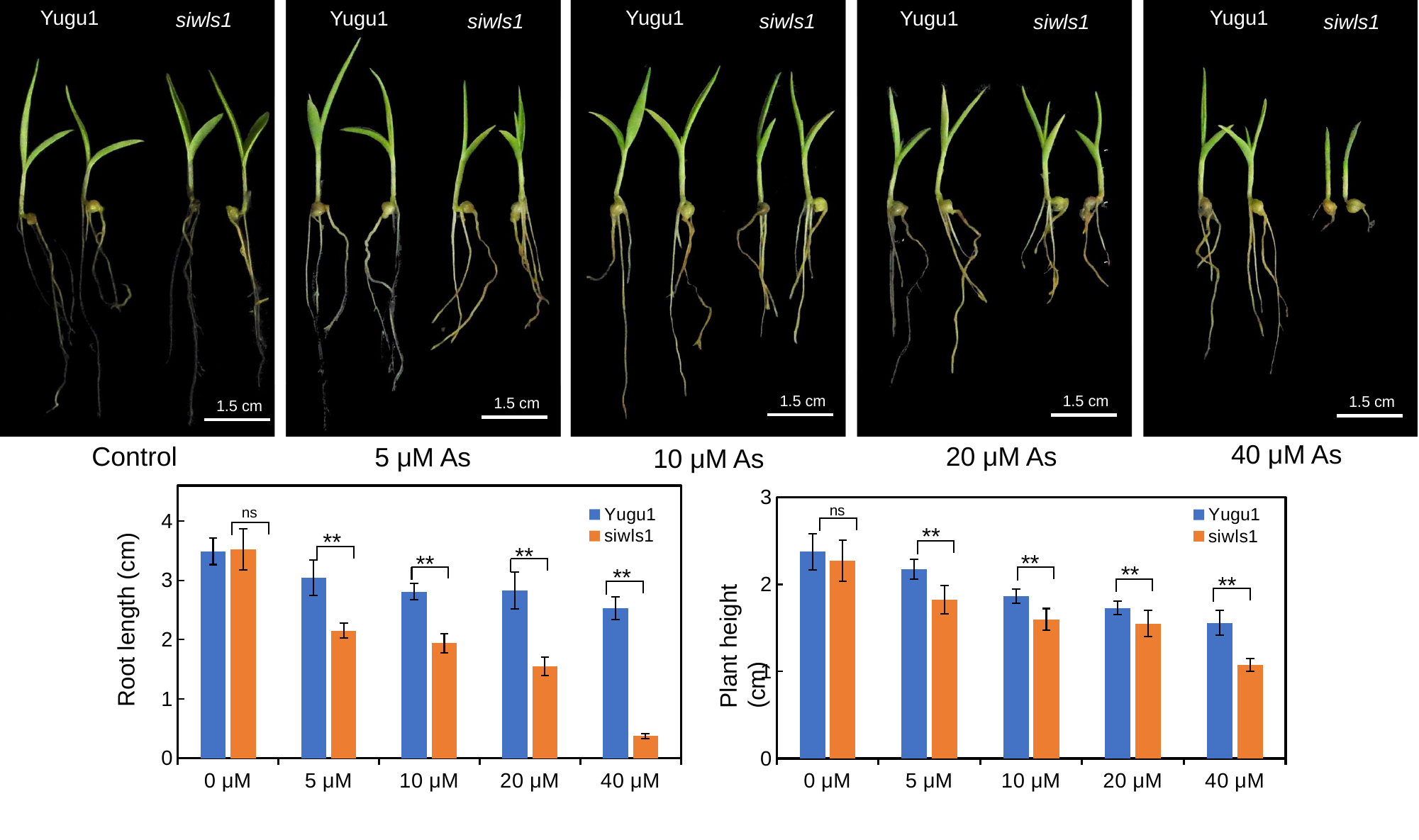

Yugu1
Yugu1
Yugu1
Yugu1
Yugu1
siwls1
siwls1
siwls1
siwls1
siwls1
1.5 cm
1.5 cm
1.5 cm
1.5 cm
1.5 cm
40 μM As
Control
20 μM As
5 μM As
10 μM As
### Chart
| Category | Yugu1 | siwls1 |
|---|---|---|
| 0 μM | 3.49 | 3.525 |
| 5 μM | 3.045 | 2.15 |
| 10 μM | 2.81 | 1.94 |
| 20 μM | 2.825 | 1.55 |
| 40 μM | 2.53 | 0.375 |
### Chart
| Category | Yugu1 | siwls1 |
|---|---|---|
| 0 μM | 2.375 | 2.275 |
| 5 μM | 2.175 | 1.825 |
| 10 μM | 1.865 | 1.6 |
| 20 μM | 1.73 | 1.55 |
| 40 μM | 1.56 | 1.075 |ns
ns
Root length (cm)
**
**
Plant height (cm)
**
**
**
**
**
**
