# Supplementary material for: Genome-Wide DNA Polymorphism Analysis and Molecular Marker Development for the Setaria italica Variety “SSR41” and Positional Cloning of the Setaria White Leaf Sheath Gene SiWLS1
Source: Front Plant Sci. 2021 Nov 11;12:743782. doi: 10.3389/fpls.2021.743782 (PMC8632227; doi:10.3389/fpls.2021.743782)
Supplement: Supplementary Figure 5 — Comparisons of plant growth between “Yugu1” and siwls1 in response to cadmium treatment. Significant differences were determined by Student’s t-test (ns = no significant difference, ∗p < 0.01, ∗∗p < 0.001). [file Presentation_5.PPTX]

## Slide 1
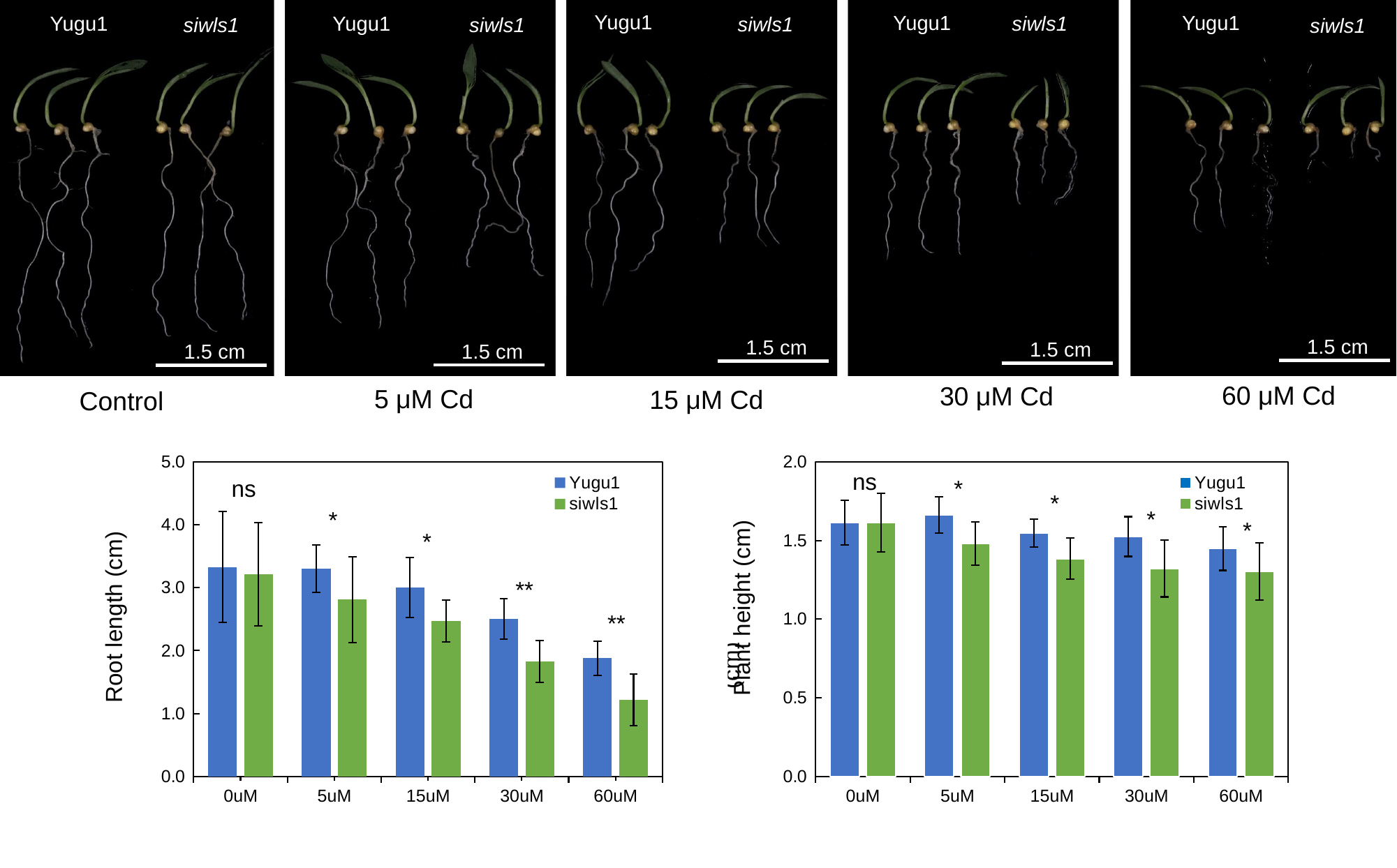

Yugu1
Yugu1
Yugu1
Yugu1
siwls1
Yugu1
siwls1
siwls1
siwls1
siwls1
1.5 cm
1.5 cm
1.5 cm
1.5 cm
1.5 cm
60 μM Cd
30 μM Cd
5 μM Cd
15 μM Cd
Control
### Chart
| Category | Yugu1 | siwls1 |
|---|---|---|
| 0uM | 3.3275000000000006 | 3.215 |
| 5uM | 3.297857142857143 | 2.811 |
| 15uM | 3.0042105263157897 | 2.469444444444444 |
| 30uM | 2.5027777777777778 | 1.8250000000000002 |
| 60uM | 1.8775000000000002 | 1.215 |
### Chart
| Category | Yugu1 | siwls1 |
|---|---|---|
| 0uM | 1.6135294117647057 | 1.61375 |
| 5uM | 1.6625000000000003 | 1.48 |
| 15uM | 1.5473684210526315 | 1.3844444444444446 |
| 30uM | 1.5249999999999997 | 1.3222222222222222 |
| 60uM | 1.4485 | 1.305 |ns
ns
*
*
*
*
Plant height (cm)
Root length (cm)
Plant height (cm)
*
*
**
**
