# Supplementary material for: Genome-Wide DNA Polymorphism Analysis and Molecular Marker Development for the Setaria italica Variety “SSR41” and Positional Cloning of the Setaria White Leaf Sheath Gene SiWLS1
Source: Front Plant Sci. 2021 Nov 11;12:743782. doi: 10.3389/fpls.2021.743782 (PMC8632227; doi:10.3389/fpls.2021.743782)
Supplement: Supplementary file 7 [file Table_2.DOCX]

**Supplementary Table S2.** **Primers designed for map-based cloning of *SiWLS1*.**

| **Primer ID** | **Chr.** | **Location** | **Forward primer** | **Reverse primer** |
| --- | --- | --- | --- | --- |
| In5-426 | Chr.5 | 42669481 | ggttactgtagcaccacattgtcaa | ggacggaagcaaggcaagca |
| In5-442 | Chr.5 | 44233870 | ttaggaggctggaaccaaacagga | gccgaggcatccatacatcttctatc |
| In5-447 | Chr.5 | 44751251 | ttgcgatgcctctgctttgtgta | aacggagcctggagacagattact |
| In5-455 | Chr.5 | 45297869 | ggttggatgggttgttaaagagttg | attaggcttaatagattcgtctcgc |
| In5-462 | Chr.5 | 46218582 | gttagtagatctggcccgcagtaaag | gcacagcgatctaagagctaggattc |
| In5-467 | Chr.5 | 46708076 | cagagcgggctggttgactt | acacatatccttacgaagacatccttg |
| SNP4636 | Chr.5 | 46384636 | accacaaattagccctccctgaatg | atcaactggtctgttcatcgcatgt |
| SNP0675 | Chr.5 | 46490675 | cctccgaattgcctcttcctctg | ccagcatagccgcttgaccttc |
| SNP0937 | Chr.5 | 46610937 | cttgcgaatttctggtaatggctatgc | aggtacttacaggagcttgagtggaa |
| siwls1 | Chr.5 | 46571596 | tcagcacagggtcatctttgggata | cgtgttcttacttgataagcagagccata |
| ln1-2 | Chr.1 | 675089 | acgaatgtatcagcgagcaagagaat | ggcagtagcagtgacacagtgag |
| ln1-8 | Chr.1 | 8719118 | catcattagcgaatggttactgtag | catgacgtaaatagcttcaagttct |
| ln1-15 | Chr.1 | 27864813 | ggtagcctcaacatttggagtttcta | gatattggttgtctaggtatcgtacag |
| ln1-17 | Chr.1 | 29536028 | ccctgctatttgaaaaatcgaaaac | gctgctcttactgatttcagattag |
| ln1-18 | Chr.1 | 40460719 | tcttctcctctttcctcgtaataac | aaaagaagaactcggagaattcaag |
| ln1-22 | Chr.1 | 41162885 | catcaaagggaagcacctgaaatgg | cggttcacctctctttactgtagcg |
| ln2-2 | Chr.2 | 1085199 | cagtgtattgaaagggcagataaaa | agttctctaccaacaataacaaagc |
| ln2-7 | Chr.2 | 6962678 | ggaacggatgttagtagcaattagatc | gattacactatacatgcacatggacac |
| ln2-11 | Chr.2 | 11412885 | gcacctattctgtttcacttacttt | ggctcacttgtagaataatggaatc |
| ln2-15 | Chr.2 | 25103187 | cataggtcttctgtttttggagttt | catctttttcgtctccctttaactt |
| ln2-19 | Chr.2 | 32193245 | gtatgcggaatcagtgagtactata | acctcttagattttgtaacgaagga |
| ln2-22 | Chr.2 | 41857700 | cacaatccacagaataaccagacaacac | ggaaggagatgacgccgacaatg |
| ln3-1 | Chr.3 | 2930596 | acaggtgaatcattgcgtcaggag | accaggcgtccgaatccagaa |
| ln3-2 | Chr.3 | 5371496 | ggcgttagatggtccctccctatt | ggctatcaatgcagcagcggtag |
| ln3-3 | Chr.3 | 5882136 | tgatcgcctggcttccacctata | gcctcaacttgtgactgtgatgtg |
| ln3-4 | Chr.3 | 6746612 | cagagcaacaatcctccctacgaat | cagcatgaatgttctggtgtcaatgat |
| ln3-12 | Chr.3 | 41168150 | cacaccagtcgtcgctgtagag | tcctcatctcctcgctcatctcg |
| ln3-14 | Chr.3 | 44045478 | gtgaacgatcaactgggatcatactt | agagaattgacaaaccaccatcga |
| ln4-1 | Chr.4 | 112965 | gatgctgctgcttgctgttcttct | tgtctgtgctataacatgaccgttcttc |
| ln4-2 | Chr.4 | 427471 | cttgccgctgcccttgttgt | gcaaatcagcccagtaccctctc |
| ln4-3 | Chr.4 | 658862 | agctcgccaagtgcagaaacc | ccaactgatgacgctcgcattaca |
| ln4-4 | Chr.4 | 1153447 | ttgatctcttcgatgctacctgaatacc | acaaccgtgagcacgcactg |
| ln4-12 | Chr.4 | 25285830 | cggcatgtaagcagtggtggaa | tggcagcggaggaagacctt |
| ln4-14 | Chr.4 | 34515535 | ccttgatcttgttgaataagcggtcag | ccgaacttcattagccaagcatcct |
| ln5-10 | Chr.5 | 14442477 | gccaagctcaagttgccacaa | ccaccactacatccgccgta |
| ln5-11 | Chr.5 | 24320045 | ttcatcatagtggcttcatcgcttgt | ctggttctgctagagtagaggaggag |
| ln5-19 | Chr.5 | 34377833 | gcttatcacactttggcactggatg | ccgctcgatactagagaacataggatg |
| ln6-3 | Chr.6 | 2573038 | cgctggagcctaatagaattgggttat | ttgttcaggatgttggactggtcag |
| ln6-7 | Chr.6 | 7517888 | agagctgagagggatttgttgattgtt | atcggtatagattatcaggactccaggtt |
| ln6-9 | Chr.6 | 9885129 | tctgtgccagcagaatcatggtaaa | ttccgcaggctacatgcaagac |
| ln6-15 | Chr.6 | 26514575 | aattagccgtaatagcgaacagagaca | ggacggatacagttactacagcagatta |
| ln6-17 | Chr.6 | 28445430 | ttggaaggcgtatctgttcttggat | ccgtcatctgttgattcagtcattgtt |
| ln6-20 | Chr.6 | 34196554 | tgaggaagaagatggcagagtcaa | gcgatgaggttaatcagtggaggaa |
| ln7-2 | Chr.7 | 1763585 | ccctttaactgtgaaatgtcagatt | ttcttccatcagatttgtgtgtaac |
| ln7-6 | Chr.7 | 13604618 | gcctctcatacctatatagcatacc | tccagcatcttactttcctttatct |
| ln7-9 | Chr.7 | 19879571 | gagccttttgagttttgacagataa | tagagaaattcttcaccttcacctt |
| ln7-15 | Chr.7 | 26919624 | aacaatcactctaacccattactct | gtcacaccatctaaatgagaacaaa |
| ln7-17 | Chr.7 | 30885002 | tctatcatacaagtcaacaggaaca | gagagggagaataaggtttttagga |
| ln7-21 | Chr.7 | 35500940 | attacaacaattcaggactcacaag | ctcctttactagttgcatcattcag |
| ln8-3 | Chr.8 | 3946443 | cgaaattaccacccgcacctacaa | gcacgctaactgccatcaacaac |
| ln8-7 | Chr.8 | 11211108 | atacctcccattttctcttaactgt | taattaactcaagccaccttctttg |
| ln8-11 | Chr.8 | 23918915 | cgtagtcctggttagtcgtagtgtac | tcgctgtggttgtagatagtgaatgtt |
| ln8-12 | Chr.8 | 25419990 | cacacctcaaccaaacagatccaaa | gcgtgacttgtgacatgcgac |
| ln8-14 | Chr.8 | 29739279 | ggagggatcaataccacaagaaatagga | gcagtagcattagcgaatacaccaa |
| ln8-21 | Chr.8 | 40307652 | aagcctcagcgaagccgact | tcctcaactgttcagcggttatttcat |
| ln9-3 | Chr.9 | 2453809 | acaacgaagaagtgagattggcagaa | ctgaagcgaacggtccgataagtt |
| ln9-4 | Chr.9 | 3642628 | atggcaagaacagcttcagtacaaca | tcatccctggattgctgggtcaa |
| ln9-5 | Chr.9 | 8082965 | cgcatcaagtgtccgtgacctt | atcaccactactccattgttgccatt |
| ln9-8 | Chr.9 | 12777496 | gcaatgcacagtaacggatctacca | catgcgttcaaaccattcttagaaagc |
| ln9-17 | Chr.9 | 44380310 | tctgaacaaggcgtgggctctta | ggcatcattactgaacgattggagaca |
| ln9-19 | Chr.9 | 45704826 | cgcgcatatcaagattcaaactttg | gacgaatggttaaatgttgcgagaa |
