# Supplementary material for: Genome-Wide DNA Polymorphism Analysis and Molecular Marker Development for the Setaria italica Variety “SSR41” and Positional Cloning of the Setaria White Leaf Sheath Gene SiWLS1
Source: Front Plant Sci. 2021 Nov 11;12:743782. doi: 10.3389/fpls.2021.743782 (PMC8632227; doi:10.3389/fpls.2021.743782)
Supplement: Supplementary file 12 [file Table_7.DOCX]

**Supplementary Table S7.** Comparison of major agronomic traits between *siwls1* and wild-type plants

| **Agronomic trait** | **Yugu1 (wild type)** | ***siwls1* (mutant)** | **Comparison** |
| --- | --- | --- | --- |
| Plant height (cm) | 30.1000 | 34.5333 | 14.73% |
| Stem diameter (mm) | 1.8933 | 1.9267 | 1.76% |
| Peduncle length (cm) | 6.7333 | 6.2167 | -7.67% |
| Flag leaf length (cm) | 20.6000 | 12.0833 | -41.34% |
| Flag leaf width (cm) | 1.4500 | 1.2367 | -14.71% |
| Panicle length (cm) | 3.6667 | 4.0000 | 9.09% |
| Panicle diameter (mm) | 8.1400 | 8.4800 | 4.18% |
| Panicle weight per plant (g) | 0.5250 | 0.5163 | -1.65% |
| 1000-grain Weight (g) | 2.5946 | 2.5726 | -0.85% |
| Grain length | 1.8764 | 2.0230 | 7.82% |
| Grain width | 1.5686 | 1.5660 | -0.16% |

Ten individuals were measured for each agronomic trait. Asterisks indicate signiﬁcant differences between WT and mutant: n=10, Welch’s two-sample t-test, *P* < 0.01.
