# Supplementary material for: Genome-Wide DNA Polymorphism Analysis and Molecular Marker Development for the Setaria italica Variety “SSR41” and Positional Cloning of the Setaria White Leaf Sheath Gene SiWLS1
Source: Front Plant Sci. 2021 Nov 11;12:743782. doi: 10.3389/fpls.2021.743782 (PMC8632227; doi:10.3389/fpls.2021.743782)
Supplement: Supplementary file 13 [file Table_8.DOCX]

**Supplementary Table S8.** Segregation of F_1_ and F_2_ populations after hybridization between *siwls1* and ‘SSR41’.

| **Combination** | **F_1_ population** | **F_2_ population** | | | | |
| --- | --- | --- | --- | --- | --- | --- |
|  |  | No. of normal green plants | No. of white sheath plants | Ratio | χ2 | α |
| *siwls1*/SSR41 | normal green | 745 | 237 | 3.13:1 | 0.347 | 0.05 |
